# Le paludisme en 2022 : aspects cliniques et thérapeutiques

**DOI:** 10.48327/mtsi.v3i2.2023.378

**Published:** 2023-06-05

**Authors:** Cécile FICKO, Pierre-Louis CONAN

**Affiliations:** 1 Service de maladies infectieuses et tropicales, Hôpital d'instruction des armées Bégin, 69 avenue de Paris, 94160 Saint-Mandé, France; 2 École du Val-de-Grâce, Paris, France

**Keywords:** Alphonse Laveran, *Plasmodium falciparum*, Paludisme, Description, Méthode, Traitement, Résistance, Alphonse Laveran, *Plasmodium falciparum*, Malaria, Description, Method, Treatment, Resistance

## Abstract

En 2022 comme en 1884, la présentation clinique du paludisme non compliqué est aspécifique : fièvre d'intensité variable, continue ou rythmée, frissons, syndrome grippal, céphalées, troubles respiratoires et digestifs. À tout moment, il peut évoluer vers une forme grave (ex-accès pernicieux ou neuropaludisme) voire létale. En relisant le *Traité des fièvres palustres* d'Alphonse Laveran, nous avons pu réaliser à quel point les observations faites alors ont permis une description méthodique et ordonnée des formes cliniques du paludisme, très proche de ce que nous pouvons, encore actuellement, observer. Aucun symptôme ou signe n'est pathognomonique de la maladie. Seule la mise en évidence des plasmodies ou « microbes du paludisme » par des méthodes directes ou immuno-chromatographiques permet la confirmation diagnostique, préalable à la mise en place d'un traitement curatif.

Sérendipité, chimie de synthèse, médecine traditionnelle, telles sont les trois méthodes qui ont permis la découverte puis la production à large échelle des antipaludiques. Sérendipité pour la quinine, chimie de synthèse pour la chloroquine, recherches menées autour de la médecine traditionnelle chinoise pour l'artémisinine et ses dérivés. Ces derniers ont marqué une véritable révolution dans la prise en charge du paludisme, tant dans ses formes non compliquées que graves. Mais, comme pour les autres antipaludiques, leur efficacité à moyen et long terme est compromise par l’émergence et la diffusion de résistances des plasmodies, notamment *P. falciparum.* Le contrôle et l’éradication du paludisme nécessitent donc la poursuite de la recherche tant dans le domaine de la prévention que celui de la thérapeutique.

La maladie si bien décrite par Alphonse Laveran n'a pas encore dit son dernier mot…

## Abstract (en)

In 2022 as in 1884, the clinical presentation of uncomplicated malaria is unspecific: fever of variable intensity, continuous or rhythmic, chills, flu syndrome, headache, respiratory and digestive disorders. At any time, it can evolve into a severe form (ex-pernicious attack or cerebral malaria) or even lethal. By reading again Alphonse Laveran's book on malarial fevers, we realized to what extent the observations made at that time allowed for a methodical and orderly description of the clinical forms of malaria, very close to what we can still observe today. No symptom or sign is pathognomonic of the disease. Only the detection of plasmodia or “malaria microbes” by direct or immuno-chromatographic methods allows for diagnostic confirmation, which is a prerequisite for the implementation of a curative treatment.

Serendipity, synthetic chemistry and traditional medicine are the three methods that led to the discovery and large-scale production of antimalarial drugs. Serendipity for quinine, synthetic chemistry for chloroquine, and research conducted around traditional Chinese medicine for artemisinin and its derivatives. The latter have marked a real revolution in the management of malaria, both in its uncomplicated and severe forms. However, as with other antimalarial drugs, its medium- and long-term efficacy is compromised by the emergence and spread of resistance in malaria parasites, particularly *P. falciparum.* The control and eradication of malaria therefore require continued research in both prevention and therapy.

The disease so well described by Alphonse Laveran has not yet said its last word…

* Actes du Colloque – Centenaire de la mort d'Alphonse Laveran. 24 novembre 2022, Paris / Proceedings of the Conference – Centenary of the death of Alphonse Laveran. 24 November 2022, Paris

Poser une question comprenant une date, ici 2022, c'est devoir y apporter une réponse s'inscrivant dans une temporalité définie. Or, si l'on s'attache au sujet du paludisme, cette temporalité est duale.

En effet, autant la littérature scientifique actuelle est riche de données concernant les antipaludiques (marqueurs moléculaires de résistance, retards de clairance parasitaire, échecs cliniques, nouvelles options ou cibles thérapeutiques) [1, 5, 15, 17] autant elle s'avère pauvre dans les descriptions cliniques du paludisme, en dehors de cadres nosologiques très spécifiques (paludisme grave, syndrome neurologique post-paludisme, etc.) [8, 10].

Parler des aspects cliniques et thérapeutiques du paludisme en 2022 constitue donc une gageure. C'est ainsi que, pour relever ce défi, nous avons voulu rendre hommage à Alphonse Laveran et à tous ceux qui ont été, ou sont impliqués dans la lutte contre cette maladie.

C'est en imaginant chausser les lunettes de notre Illustre Ancien et en nous plongeant dans le *Traité des fièvres palustres* que nous avons réappris ce que nous lui devions concernant la description clinique du paludisme.

En effet, outre les descriptions de ce qu'A. Laveran nommait les « microbes du paludisme », son ouvrage, empreint de méthode et de rigueur, comprend une description systématique et ordonnée des aspects cliniques de cette parasitose [7]. Les progrès réalisés alors dans la classification des fièvres palustres sont immenses. Au chaos des fièvres, avec des cadres multiples (fièvre pernicieuse délirante, comateuse, gastralgique, cholériforme, bilieuse, etc.), A. Laveran répond par la méthode. Ses centaines d'observations faites en Algérie, dont beaucoup sont décrites individuellement, lui permettent même de faire part de la « monotonie » des manifestations cliniques du paludisme, responsables d’« ennui dans les services cliniques des pays chauds »…

En 1884 déjà, aucun signe clinique n’était spécifique du paludisme, comme cela est le cas pour une autre fièvre, typhoïde celle-ci. En 1884 déjà, cette maladie était trompeuse, avec des tableaux des plus frustes aux plus sévères, pouvant conduire au décès. Ceci nous est d'ailleurs rappelé, aujourd'hui, tant par la Société de pathologie infectieuse de langue française (SPILF) que par l'Organisation mondiale de la santé (OMS) : symptômes et signes cliniques aspécifiques, rendant le diagnostic parasitologique indispensable [13, 18]. En 1884 déjà, A. Laveran rassemblait ses descriptions cliniques en quatre catégories : « fièvres intermittentes simples, fièvres continues palustres, fièvres intermittentes ou continues compliquées d'accidents graves dits pernicieux, cachexie palustre ».

Ainsi, ce que nous avons appris de nos Maîtres et ce que nous enseignons à nos élèves, nous le devons pour beaucoup à Alphonse Laveran : le continuum physiopathologique et clinique entre accès simples (avec fièvre intermittente ou continue) et accès graves (ex-pernicieux) et la description du paludisme viscéral évolutif (ex-cachexie palustre), apanage des sujets vivant en zone d'endémie. Certaines formes particulières (splénomégalie malarique hyper-réactive, fièvre bilieuse hémoglobinurique) ou survenant sur des terrains particuliers (femmes enceintes) ne seront décrites que plus tard.

Mais le paludisme, dans sa description clinique, est donc tel qu'il était en 1884 : aspécifique toujours, trompeur parfois, grave si on le laisse nous piéger.

Sur le plan thérapeutique, sans vouloir nous attacher à dresser un tableau exhaustif des antipaludiques d'hier et d'aujourd'hui, nous avons souhaité mettre en avant trois concepts dans leurs processus respectifs de recherche et développement : sérendipité, chimie de synthèse et compréhension de la médecine traditionnelle.

L'utilisation de la quinine est un exemple très démonstratif de sérendipité : dès le xvii^e^ siècle, les vertus antipyrétiques de l’écorce de quinquina étaient connues en Amérique du Sud, notamment vis-à-vis de la fièvre tierce. Elle a ensuite été introduite en Europe, pour être utilisée comme traitement des fièvres intermittentes. Ce n'est qu'au xix^e^ siècle que Caventou et Pelletier en extraient le principe actif, la quinine, et qu'elle se voit produite à large échelle. Dans son traité, A. Laveran notait son efficacité sur les fièvres palustres, tant sur le plan clinique (guérison même de certains accès pernicieux) que microscopique (disparition sous traitement des « microbes du paludisme »). Et c'est au xx^e^ siècle que les modes d'action de ce schizonticide érythrocytaire ont été mis en évidence (inhibition de la protéase dégradant les acides aminés de l'hémoglobine et de la polymérisation de l'hème, indispensables au cycle des plasmodies) [2]. Cependant, bien qu'ayant constitué un progrès thérapeutique majeur, sa marge thérapeutique étroite, exposant à une potentielle toxicité cardiaque et neurologique, a imposé la poursuite de la recherche sur les antipaludiques curatifs.

C'est à compter des années 1920 que, via la chimie de synthèse, sont menées des recherches sur des dérivés moins toxiques que la quinine. La sontochine, amino-4-quinoléïne, schizonticide érythrocytaire, est créée en 1936. Après des essais cliniques au début des années 1940, elle est rebaptisée chloroquine en 1946, et mise sur le marché à partir de 1949-1950 [6]. Mais, outre sa marge thérapeutique étroite, exposant là encore à une toxicité cardiaque et neurologique, l'apparition puis la diffusion progressive de résistances, notamment chez *P. falciparum,* grève l'efficacité de cette molécule tant concernant la prise en charge individuelle que le contrôle collectif du paludisme à l’échelle mondiale.

De nombreuses autres molécules et associations thérapeutiques sont progressivement découvertes, synthétisées et mises sur le marché [16]. Mais, malgré cela, le paludisme reste un véritable problème de santé publique en zone d'endémie.

C'est en 1967, en Chine, que sont lancés de vastes programmes de recherche sur les antipaludiques, du fait du lourd tribut payé au paludisme lors de la guerre du Vietnam. Deux méthodes sont utilisées en parallèle : criblage de molécules issues de la chimie de synthèse d'une part et recours à la médecine traditionnelle chinoise d'autre part. Dans le cadre de ce projet, 523, Youyou Tu et son équipe s'attachent à analyser de nombreuses préparations traditionnelles. Une plante, l'armoise annuelle ou *Artemisia annua,* ou quinghao, finit par retenir son intérêt en montrant des propriétés antipaludiques prometteuses. Là encore, c'est grâce à la lecture de traités de médecine traditionnelle datant du iii^e^ siècle qu'est redécouverte la méthode d'extraction à froid du principe actif issu de cette plante, qui démontrera ensuite rapidement son efficacité clinique. L'artémisinine, ou quinghaosu, voyait le jour en 1972 [9]. Parasiticide puissant et d'action très rapide, sa demi-vie courte impose toutefois son association à une molécule de demi-vie longue afin de prévenir l’émergence de plasmodies résistantes. L'artémisinine et ses dérivés constituent une véritable révolution thérapeutique dans la prise en charge du paludisme, notamment à *P. falciparum.* Après un plaidoyer en 2001 quant à l'intérêt de l'artémisinine, les combinaisons thérapeutiques sont recommandées par l'OMS comme 1^re^ ligne de traitement du paludisme non compliqué depuis 2006 [11]. L'artésunate intra-veineux, après avoir démontré une efficacité bien supérieure à la quinine dans des études menées en zone d'endémie, est quant à lui indiqué comme traitement du paludisme grave depuis 2010 [3, 4, 12]. Pour sa découverte, Youyou Tu reçoit le prix Nobel de médecine en 2015.

Médicaments efficaces, bien tolérés, amélioration du diagnostic avec diffusion des tests de diagnostic rapide en zone d'endémie, déploiement à large échelle des moustiquaires imprégnées d'insecticide, recherche vaccinale : les années 2000 voient germer l'espoir du contrôle du paludisme.

Mais des résistances à l'artémisinine et ses dérivés auraient émergé dès 2001 en Asie du Sud-Est, pour être prouvées en 2008. Retards de clairance parasitaire, échecs cliniques et parasitologiques, cette problématique diffuse ensuite progressivement en zone d'endémie, notamment en Afrique subsaharienne, même si elle n'y constitue pas encore un problème de santé publique [14, 16].

L’éternelle course de vitesse contre les plasmodies n'est donc pas terminée. Développement de trithérapies antipaludiques, mise en évidence de nouvelles cibles thérapeutiques, de pistes prometteuses en matière de prévention (vaccins, anticorps monoclonaux) : la partie est loin d’être gagnée, mais la lutte continue afin de contrôler, puis d’éradiquer cette maladie qu'a si bien décrite Alphonse Laveran.

## Contribution Des Auteurs

Les auteurs ont vu et approuvé le manuscrit et ont contribué de manière significative au travail.

## Liens D'intérêt

Les auteurs ne déclarent aucun conflit d'intérêt.

## References

[1] Arya A, Kojom Foko LP, Chaudhry S, Sharma A, Singh V (2021). Artemisinin-based combination therapy (ACT) and drug resistance molecular markers: A systematic review of clinical studies from two malaria endemic regions - India and sub-Saharan Africa. Int J Parasitol Drugs Drug Resist.

[2] Chast F (2020). La découverte de la quinine par Joseph Pelletier et Joseph B. Caventou (1820). Revue de biologie médicale.

[3] Dondorp A, Nosten F, Stepniewska K, Day N, White N, South East Asian Quinine Artesunate Malaria Trial (SEAQUAMAT) group (2005). Artesunate versus quinine for treatment of severe *falciparum* malaria: a randomised trial. Lancet.

[4] Dondorp AM, Fanello CI, Hendriksen IC, Gomes E, Seni A, Chhaganlal KD, Bojang K, Olaosebikan R, Anunobi N, Maitland K, Kivaya E, Agbenyega T, Nguah SB, Evans J, Gesase S, Kahabuka C, Mtove G, Nadjm B, Deen J, Mwanga-Amumpaire J, Nansumba M, Karema C, Umulisa N, Uwimana A, Mokuolu OA, Adedoyin OT, Johnson WB, Tshefu AK, Onyamboko MA, Sakulthaew T, Ngum WP, Silamut K, Stepniewska K, Woodrow CJ, Bethell D, Wills B, Oneko M, Peto TE, von Seidlein L, Day NP, White NJ, AQUAMAT group (2010). Artesunate versus quinine in the treatment of severe *falciparum* malaria in African children (AQUAMAT): an open-label, *falciparum* trial. Lancet.

[5] Hamaluba M, van der Pluijm RW, Weya J, Njuguna P, Ngama M, Kalume P, Mwambingu G, Ngetsa C, Wambua J, Boga M, Mturi N, Lal AA, Khuroo A, Taylor WRJ, Gonçalves S, Miotto O, Dhorda M, Mutinda B, Mukaka M, Waithira N, Hoglund RM, Imwong M, Tarning J, Day NPJ, White NJ, Bejon P, Dondorp AM (2021). Arterolane-piperaquine-mefloquine versus arterolane-piperaquine and artemether-lumefantrine in the treatment of uncomplicated *Plasmodium falciparum* malaria in Kenyan children: a single-centre, open-label, randomised, non-inferiority trial. Lancet Infect Dis.

[6] Krafts K, Hempelmann E, Skórska-Stania A (2012). From methylene blue to chloroquine: a brief review of the development of an antimalarial therapy. Parasitol Res.

[7] Laveran A (1884). Traité des fièvres palustres avec la description des microbes du paludisme. https://archive.org/details/traitdesfivr00lave.

[8] Lebut J, Mourvillier B, Argy N, Dupuis C, Vinclair C, Radjou A, de Montmollin, E, Sinnah F, Patrier J, Le Bihan C, Magalahes E, Smonig R, Kendjo E, Thellier M, Ruckly S, Bouadma L, Wolff M, Sonneville R, Houzé S, Timsit JF (2020). Changes in the clinical presentation and outcomes of patients treated for severe malaria in a referral French university intensive care unit from 2004 to 2017. Ann Intensive Care.

[9] Mazier D, Thellier M, Youyou Tu, de Mao Zedong au Prix Nobel–Prix Nobel de Médecine 2015: William C (2016). Campbell, Satoshi Ōmura et Youyou Tu. Med Sci (Paris).

[10] Muigg V, Maier MI, Kuenzli E, Neumayr A (2021). Delayed cerebellar ataxia, A rare post-malaria neurological complication: Case report and review of the literature. Travel Med Infect Dis.

[11] OMS (2006). Guidelines for the treatment of malaria. Organisation mondiale de la Santé.

[12] OMS (2010). Guidelines for the treatment of malaria. Organisation mondiale de la Santé.

[13] OMS (2021). Lignes directrices de l'OMS sur le paludisme, 16 février 2021. Organisation mondiale de la Santé.

[14] OMS (2021). World malaria report 2021. Organisation mondiale de la Santé.

[15] Peto TJ, Tripura R, Callery JJ, Lek D, Nghia HDT, Nguon C, Thuong NTH, van der Pluijm RW, Dung NTP, Sokha M, Van Luong V, Long LT, Sovann Y, Duanguppama J, Waithira N, Hoglund RM, Chotsiri P, Chau NH, Ruecker A, Amaratunga C, Dhorda M, Miotto O, Maude RJ, Rekol H, Chotivanich K, Tarning J, von Seidlein L, Imwong M, Mukaka M, Day NPJ, Hien TT, White NJ, Dondorp AM (2022). Triple therapy with artemether-lumefantrine plus amodiaquine versus artemether-lumefantrine alone for artemisinin-resistant, uncomplicated *falciparum* malaria: an open-label, randomised, multicentre trial. Lancet Infect Dis.

[16] Pradines B, Dormoi J, Briolant S, Bogreau H, Rogier C (2010). La résistance aux antipaludiques. Rev Francoph Lab.

[17] Rasmussen C, Ringwald P (2022). Is triple artemisinin-based combination therapy necessary for uncomplicated malaria?. Lancet Infect Dis.

[18] Société de pathologie infectieuse de langue française (SPILF) (2023). Prise en charge et prévention du paludisme d'importation. Mise à jour 2017 des recommandations pour la pratique clinique 2007. Consulté le.

